# The comparative effect of ionophore and thymol on growth performance, intestinal histopatholgy and cytokine expression in broilers challenged with mixed *Eimeria* species

**DOI:** 10.1186/s12917-026-05794-0

**Published:** 2026-08-06

**Authors:** Pratik G Kokate, Suresh Gulabrao Jadhao, Pranav P Deshmukh, Sunil W Kolte, Mahesh Gupta, Shubham P Mandhale, Mukund M Kadam, Sonali D Borkar, Snehil Gupta

**Affiliations:** 1 Nagpur Veterinary College, Maharashtra Animal and Fishery Sciences University, Nagpur, 440006 India; 2 ICAR-National Institute of Abiotic Stress Management, Baramati, Pune, 413115 India; 3 Lala Lajpat Rai University of Veterinary and Animal Sciences, Hisar, Haryana 125004 India; 4 Department of Veterinary Parasitology, Nagpur Veterinary College, MAFSU, Nagpur, 440006 India

**Keywords:** Coccidiosis, *Eimeria*, Ionophore antibiotics, Pro-inflammatory cytokines

## Abstract

**Background:**

Coccidiosis, caused by seven valid species of *Eimeria*, poses a significant challenge to the poultry industry. There is a growing interest in natural feed additives to mitigate the detrimental effects of coccidial infections in poultry farming. This study investigates the comparative effects of ionophore antibiotics (salinomycin and monensin) and phytochemical (thymol) on growth performance, intestinal morphology, and mucosal cytokine response in broilers challenged with sporulated oocysts of multiple *Eimeria* species at 21 day of age of birds. A total of 225 one-day-old broiler chicks were randomly assigned to five groups, group (G) A (untreated unchallenged; (Negative control); G B (untreated challenged; (Positive control), G C, D and E; three challenged groups, each of which are supplemented with salinomycin, monensin and thymol respectively. Performance parameters, oocyst per gram (OPG), cytokine expression (interleukin-1 beta (IL-1β) and interleukin 6 (IL-6) and histopathological assessments were conducted on days 21(0 day post infection(dpi)), 28 (7 dpi), and 42 (21 dpi).

**Results:**

The PC group exhibited high mortality (15.56%) and the highest OPG counts at 14 and 21 dpi, while thymol supplementation resulted in a slower decline in OPG counts at 14 dpi but showed significant reduction by 21 dpi. These findings indicate that, although thymol was less potent than ionophore anticoccidials under the conditions tested but beneficial as a non-chemical alternative molecule for coccidiosis management. Salinomycin and monensin groups had zero OPG counts at both time points. Significant differences in feed intake and feed conversion ratio (FCR) were observed, with salinomycin demonstrating better FCR performance than monensin and thymol. At 7-dpi (28 days of age), the positive control group exhibited marked upregulation of the pro-inflammatory cytokines IL-1β and IL-6 in both the jejunum and spleen compared with the negative control. In contrast, all anticoccidial-treated groups showed significantly reduced cytokine expression. Among the treatments, thymol supplementation resulted in the lowest jejunal IL-1β expression, whereas salinomycin and monensin produced comparable suppression of IL-6 expression in both tissues. At 21-dpi, cytokine expression in all treated groups had returned close to basal levels, indicating effective resolution of inflammation. Histopathological examination further demonstrated that salinomycin and monensin markedly reduced intestinal lesions and parasitic developmental stages, whereas thymol improved intestinal architecture by reducing inflammatory cell infiltration.

**Conclusion:**

These results indicate that salinomycin and monensin exhibited superior anticoccidial efficacy by effectively reducing oocyst shedding, preserving intestinal histopathology, and attenuating inflammatory cytokine expression, whereas thymol exerted beneficial immunomodulatory and histopathological effects with comparatively lower anticoccidial efficacy, highlighting its potential as a natural alternative to commercial anticoccidials.

**Supplementary Information:**

The online version contains supplementary material available at https://doi.org/10.1186/s12917-026-05794-0.

## Background

Poultry coccidiosis is economically most important protozoan disease, caused by seven valid highly site-specific and host-specific *Eimeria* species, infecting different regions of the intestinal tract, resulting in an annual economic loss of about $14.5 billion [1]. Among all valid species, *Eimeria tenella* is the most prevalent and pathogenic, causing severe diarrhea, high morbidity and mortality by damaging intestinal epithelial cells, impairing nutrient absorption, reducing feed efficiency, and inducing substantial blood loss, thereby highlighting the need for effective control measures to sustain poultry health and productivity [2]. In addition to *E. tenella*, other pathogenic species including *E. necatrix*,* E. brunetti*,* E. maxima*,* E. acervulina*,* E. mitis* and *E. praecox* contribute substantially to avian coccidiosis. Among these, *E. necatrix* and *E. brunetti* are considered highly pathogenic, causing severe hemorrhagic enteritis and considerable mortality, whereas *E. maxima* and *E. acervulina* are moderately pathogenic and primarily associated with reduced weight gain and poor feed conversion efficiency. In contrast, *E. mitis* and *E. praecox* are relatively less pathogenic but can cause significant subclinical losses through impaired nutrient absorption and growth performance, particularly in mixed infections [3–5].

For many years, poultry coccidiosis is locally managed in tropical countries like India using commercial anticoccidials as feed additives. however, concerns regarding anti-coccidial drug resistance, drug residues, regulatory restrictions, and the emergence of drug-resistant *Eimeria* strains have highlighted the need for alternative eco-friendly and sustainable approaches [6]. Ionophores are polyether antibiotic feed additives widely used for the prevention and control of avian coccidiosis. Produced primarily by *Streptomyces* spp., they exert their anticoccidial activity by facilitating the transmembrane transport of cations such as Na⁺, K⁺ and Ca²⁺, thereby disrupting ionic homeostasis in *Eimeria* parasites and ultimately causing parasite death [7, 8]. The commercially important ionophore anticoccidials include salinomycin, monensin, narasin, lasalocid and maduramicin. Owing to their broad-spectrum anticoccidial efficacy, growth-promoting properties and relatively slower development of resistance than many synthetic anticoccidials, ionophores have remained the cornerstone of coccidiosis control programmes in the poultry industry worldwide [9, 10].

A preliminary field survey conducted among poultry farmers and veterinarians in the poultry belt of Nagpur, Maharashtra, India, identified salinomycin and monensin as the most commonly used ionophore anticoccidials in commercial broiler production. To the best of our knowledge, this is the first report documenting the pattern of anticoccidial usage in this region. The survey interview schedule and summary of responses are provided as Supplementary Material (Supplementary Fig. S1). Salinomycin and monensin were therefore selected as the representative ionophore anticoccidials for evaluation in the present study because of their widespread field use and established efficacy against *Eimeria* infections.

In response to the increasing consumer demand for safe and organic poultry products, natural anticoccidial alternatives, particularly phytogenic compounds, have attracted considerable attention because of their low toxicity, wide availability and suitability as feed additives [11].

Among phytochemicals, essential oils have been widely investigated for coccidiosis control and major bioactive constituents of these selective essential oils are thymol, eugenol and carvacrol [12]. Of these, thymol has recently gained pharmaceutical industry attention due to antioxidant, anti-inflammatory, antibacterial, antifungal, and antineoplastic activities, largely by suppression of pro-inflammatory cytokines and inflammatory mediators [13, 14]. In poultry coccidiosis, intestinal pathogenesis is mainly contributed by inflammatory response mediators generated in response to *Eimeria* infection [15]. Accordingly, the present study aimed to compare the efficacy of commonly used ionophore antibiotics in the field with thymol to provide a phyto-alternative feed additive for sustainable poultry farming and management of coccidiosis in field conditions. Further, the two commonly used ionophore antibiotics in the field will be compared for their performance in the broiler industry. As pathogenesis in coccidiosis is mainly inflammation driven, therefore, the impact of these drugs on pro-inflammatory cytokines will also be investigated.

## Materials and methods

### Procurement of materials

The commercial anticoccidial drugs, Monensin Sodium, were obtained from Elanco™, Greenfield, Indiana. Salinomycin premix (12%) was purchased from Shandong Lukang Shelile Pharmaceutical Co., Ltd., China. Thymol-Phyto (25%) was sourced from Imkuraq Animal Health™, Hyderabad, Telangana, India.

### Source of *Eimeria* species oocysts

*Eimeria* oocysts were collected from the feces of naturally infected broilers from poultry farms surrounding Nagpur (India). The presence of oocysts was confirmed by observation of oocysts under compound microscope. The oocysts were morphologically identified as *Eimeria tenella*,* E. mitis* and *E. acervulina* under digital microscope after sporulation in 2.5% potassium dichromate solution. Molecular confirmation of species identity was not performed; therefore, species identification was based solely on the morphological characteristics of sporulated oocysts, which is acknowledged as a limitation of the present study. The morpholocal identification of *Eimeria* oocysts are given in supplementary Fig. S2. The inoculum consisted of a mixed population of these sporulated oocysts. Individual *Eimeria* species were identified morphologically but were not quantified separately; therefore, the challenge dose was standardized based on the total oocyst count. The oocysts were isolated using the standard sedimentation technique described by [16]. The collected oocysts were sporulated by incubation in 2.5% K_2_Cr_2_O_7_ solution in petri dishes for 72 h at 25–28 °C, following the protocol described by Conway and McKenzie [3]. A continuous supply of oocysts maintained by propagating the sporulated oocysts in 12 broiler birds (15-d old). The sporulated oocysts were separated and concentrated in 25 mL solution, each ml containing 20,000 (2 × 10^4^) sporulated oocyst of mixed *Eimeria* species. The 12 birds used for oocyst propagation were challenged orally on 15th day with 2 mL of solution containing approximately 4 × 10^4`^ sporulated mixed *Eimeria* spp. oocysts [3, 17]. The faeces of these 12 broiler birds were collected from 4-dpi to 10-dpi and oocysts were concentrated by the standard floatation technique. The oocysts were sporulated in the laboratory by standard protocol. A single homogeneous inoculum was prepared and thoroughly mixed before the administration to ensure comparable exposure among all challenged groups. This inoculum was used as a source of oocysts for experimental infection in the current study.

### Ethics and consent

The experiment was approved by the Institutional Animal Ethics Committee of Nagpur Veterinary College with Approval Number NVC/IAEC/03/2023.

### Experimental design, birds and management

A total of 225, day-old unsexed Cobb 430 commercial broiler chicks were purchased from Sri Rajeshwara Hatcheries Pvt Ltd., Hederabad, India, were randomly divided into five treatment groups, viz. Group A (basal corn–soybean diet without probiotics or coccidiostat) [negative control], Group B (basal diet without probiotics or coccidiostat) [positive control], Group C (basal diet + salinomycin 50 ppm) [18], Group D (basal diet + monensin 100 ppm) [19], and Group E (basal diet + thymol 40 ppm) [20]. The supplementation level was selected based on previous evidence demonstrating the anticoccidial efficacy and safety of thymol and supported by studies reporting its beneficial effects in broiler chickens [21–25]. All birds were offered a basal corn–soybean diet formulated without probiotics or coccidiostats. The basal diet served as the control diet and was supplemented with salinomycin, monensin, or thymol in the respective treatment groups according to the experimental design. Each group consisted of three replicates with 15 chicks per replicate, and the birds were reared for 42 days under standard deep-litter management with ad libitum feed and water. Representative faecal samples were collected from each replicate and examined daily by the sedimentation technique to confirm the absence of *Eimeria* oocysts before challenge, and all birds were vaccinated against Newcastle disease on days 7 and 21.

On day 21, all groups except group A were challenged with a mixed sporulated *Eimeria* inoculum at a dose of 4.0 × 10^5^ oocysts/m² of litter, applied by uniform spraying onto the litter, following a previously described floor-pen litter challenge model [25]. A litter-based mixed *Eimeria* spp. challenge was selected in the present study to simulate field exposure conditions, in which birds acquire infection through ingestion of contaminated litter [26, 27]. Birds were then observed for 21 days post-infection (dpi) to evaluate growth performance. The basal diet composition is presented in Table 1. The basal diets were formulated to meet the nutrient requirements of broiler chickens according to the Bureau of Indian Standards (BIS, 2007) recommendations. The nutrient composition presented in Table 1 was calculated from the ingredient composition using standard nutrient values provided by BIS (2007) [28].

**Table 1:** Ingredient composition of basal diet and nutrient levels

Feed Ingredients	Pre-Starter (0–7 days)	Starter (8–21 days)	Finisher (21–42 days)
Maize	57.50	59.00	61.00
Soybean meal	36.40	33.45	30.00
Vegetable oil	1.75	3.20	4.50
Monocalcium phosphate	1.50	1.40	1.50
Limestone powder	1.40	1.50	1.65
Salt	0.30	0.30	0.30
Trace Min. Premix	0.05	0.05	0.05
Vitamin Premix	0.05	0.05	0.05
DL-Methionine	0.30	0.35	0.30
L-Lysine	0.30	0.25	0.25
L-Threonine	0.15	0.15	0.10
Choline Chloride	0.15	0.15	0.15
Toxin binder	0.15	0.15	0.15
Total	100	100	100
Nutrient levels (%)			
Dry matter	88.9	89.3	90.2
Crude protein	23.4	21.8	20.1
Ether extract	4.09	5.20	6.30
Crude fibre	3.68	3.50	3.32
N.F. E^1^	62.61	63.18	63.79
Total ash	6.22	6.32	6.49
ME^2^ (Kcal/kg)	3154	3252	3280

The basal diet formulation was identical for all experimental groups. Anticoccidial supplementation was incorporated only in the designated treatment groups according to the experimental design, namely salinomycin in Group C, monensin in Group D, and thymol in Group E. Groups A (negative control) and B (positive control) received the basal diet without anticoccidial supplementation.^1^NFE: Nitrogen free extract; ^2^ME: Metabolic energy

### Growth performance

The birds and feed were weighed weekly to determine phase-wise growth performance. To determine the growth performance, the average body weight gain (ABWG), average cumulative feed consumption (ACFC), and average cumulative FCR, was recorded during starter (days 1–21) and finisher (days 22–42) phase using a precise digital scale [29, 30]. Feed intake was calculated weekly based on the amount offered and leftovers for each treatment group. Feed conversion ratios (FCR) were computed weekly. Mortalities were noted, with dead birds weighed and subjected to post-mortem examinations. All birds were challenged simultaneously on day 21.

### Oocyst per gram (OPG) of feces count

The OPG (oocyst per gram of feces) count was done weekly. The samples were collected from each replicate pen rather than from individual birds, as the replicate pen was considered as experimental unit for oocysts quantification. The samples were collected in sterile wide-mouth containers and were brought to the laboratory and subjected to standard parasitological faecal sedimentation procedures. The OPG was done by the standard McMaster chamber method. Fecal samples from each replicate were collected from each replicate pen at 0, 7, 14, and 21 days post-infection and examined for OPG estimation using the McMaster counting technique. Samples yielding no detectable oocysts within the detection limit of the counting chamber were recorded as zero OPG. To confirm negative results, repeated microscopic examinations were performed, and no *Eimeria* oocysts were detected in any samples collected from the negative control group throughout the experimental period.

### Intestinal morphology

On days 21, 28 and 42, six birds from each group (2 birds per replicate) were sacrificed after euthanizing by electrical stunning and followed by exsanguination in accordance with the approved Institutional Animal Ethics Committee (IAEC) protocol. Electrical stunning was used to induce rapid unconsciousness and minimize the pain and distress before exsanguination, while exsanguination facilitated adequate tissue perfusion for optimal histopathological evaluation. The intestinal tissues were collected to investigate the intestinal lesions. Further, 2 cm segments in the middle of the duodenum, jejunum, ileum and caecum were collected in 10% neutral buffered formalin solution for histopathological examination. The samples were sequentially dehydrated with 70%, 90%, and 100% ethyl alcohol and cleared with xylene before embedding in paraffin blocks. The blocks were cut into standard size of 5 μm long pieces using a rotatory microtome, followed by staining with hematoxylin and eosin (H&E staining).

### Relative mRNA expression of pro-inflammatory cytokines

In order to examine the cytokine expression of pro-inflammatory cytokines (IL-1β and IL-6) released using early invasion of coccidian oocyst in intestinal epithelial cells, tissue samples (50 mg) were taken from the spleen and jejunum of six birds in each treatment group on days 21, 28, and 42. The total RNA was extracted with TRIzol reagent (Thermo Fisher Scientific, Wilmington, DE). The RNA was quantified via spectrophotometry, and its quality was assessed through the OD260/OD280 ratio. The integrity of extracted RNA was confirmed by observing 18 S and 28 S ribosomal RNA bands on a 1% agarose gel. The 18 S and 28 S ribosomal RNA bands were used solely to confirm RNA integrity and were not used for gene expression normalization. Subsequently, 1 µg of total RNA from each sample was reverse transcribed into cDNA via the GoScript™ reverse transcription kit (Promega, USA) following the manufacturer’s instructions. The GoTaq qPCR SYBR Master Mix (Promega, USA) was used to carry out real-time quantitative polymerase chain reaction (qRT-PCR) on an Insta Q 96 real time PCR Design & Analysis system (Himedia, India). The reactions were performed in a 20 µL mixture that included 10.0 µL of 2× Gotaq qPCR master mix, 1.0 µL of cDNA, 0.5 µL of each primer (10 mmol/L), and 8.0 µL of RNase-free water. For PCR, the thermocycling procedure lasted 2 min at 95 °C, followed by 40 cycles of denaturation at 95 °C for 10 s, annealing/extension at 60 °C for 35 s, and a final melting curve analysis to check the purity of the PCR product. The sequence of primers used in this study are shown in Table 2. Primers for IL-1β gene were adopted from Li [31], while IL-6 primers were custom designed using Primer3 tool of NCBI using accession number NM_204628 of *Gallus gallus* origin. Avian β-actin was used as the internal reference (House keeping) gene for normalization of target gene expression, and relative mRNA expression levels were normalized to those of avian β-actin by 2^−∆∆Ct^ method [32].

**Table 2:** Primer sequences used for q-PCR

Gene	Sequences 5′-3′	Efficiency (%)	Amplicon length (bp)	Annealing Temperature (°C)	Reference
ACTB	F: GAGAAATTGTGCGTGACATCA R: CCTGAACCTCTCATTGCCA	108.2	152	60	Wu et al., (2021) [33]
IL-1β	F: ACTGGGCATCAAGGGCTA R: GGTAGAAGATGAAGCGGGTC	98	131	60	Li et al., (2018) [31]
IL-6	F: AGGGCCGTTCGCTATTTGAA R: CAGAGGATTGTGCCCGAACT	102	72	60	NM_204628

*ACTB* β-actin, *IL* Interleukin, *F *forward primer, *R *reverse primer

### Statistical analysis

The data collected in this study were statistically analyzed using IBM SPSS Statistics software for Windows, Version 21.0 (IBM Corp., Armonk, NY, USA). Data were analyzed using one-way analysis of variance (ANOVA) followed by Tukey’s post hoc multiple comparison test using SPSS, version 21.0. Each sampling time point was analyzed independently; therefore, no additional correction across time points was applied. Differences were considered statistically significant at *P* < 0.05. The results were indicated as mean ± standard error (SE).

## Results

### Effect on growth performance

The growth performance of broiler chickens during the pre-coccidial challenge phase, encompassing average body weight gain, average feed consumption, and feed conversion ratio (FCR), following dietary supplementation with different anticoccidial agents from day 1 to day 21, is presented in Table 3. During this pre-challenge period, statistical evaluation indicated that supplementation of the basal diet with salinomycin, monensin or thymol did not exert any significant influence (*P* > 0.05) on the measured growth performance parameters when compared with broilers fed with the basal diet. The influence of various anticoccidial agents on growth performance was further assessed during the post-challenge phase spanning from day 21 to day 42, and the corresponding results are summarized in Table 3. Statistical analysis revealed that dietary supplementation with different anticoccidial agents did not result in significant differences (*P* > 0.05) in body weight gain among the treatment groups when compared to birds maintained on the basal diet.

**Table 3:** Performance of broilers from 0–42 day

Parameters	Dietary Treatment					*P* Value
	Group A (Negative control)	Group B (Positive control)	Group C (Salinomycin)	Group D (Monensin)	Group E (Thymol)	
ABWG (g/bird)						
D 1–21	776.08 ± 28.51	798.64 ± 19.18	843.56 ± 32.10	813.84 ± 36.44	808.87 ± 18.99	0.34
D 22–42	1809.90 ± 22.72	1722.22 ± 38.41	1748.34 ± 50.63	1764.67 ± 49.96	1753.33 ± 35.75	0.66
D 1–42	2585.98 ± 5.69	2520.87 ± 30.93	2591.89 ± 43.74	2578.51 ± 16.96	2562.20 ± 27.30	0.44
ACFI (g/bird)						
D 1–21	1033.33 ± 12.02	1030.00 ± 32.15	1053.33 ± 53.64	1033.33 ± 31.80	1023.33 ± 12.02	0.97
D 22–42	3317.08 ± 7.39	3449.25 ± 47.75	3348.90 ± 30.16	3377.43 ± 52.48	3407.33 ± 30.53	0.56
D 1–42	4340.41^c^ ± 9.58	4489.25^ab^ ± 19.47	4392.23^bc^ ± 32.12	4410.76^bc^ ± 21.91	4425.33^ab^ ± 24.19	< 0.05
Average C-FCR						
D 1–21	1.26 ± 0.04	1.22 ± 0.03	1.19 ± 0.02	1.20 ± 0.01	1.20 ± 0.03	0.83
D 22–42	1.83^b^± 0.03	2.00^a^± 0.02	1.91^b^± 0.03	1.91^b^± 0.03	1.94^b^± 0.04	< 0.05
D 1–42	1.65^b^ ± 0.02	1.75^a^ ± 0.02	1.67^b^ ± 0.03	1.68^b^ ± 0.02	1.69^b^ ± 0.03	< 0.05
Mortality (%)	0.00^c^	15.56^a^	0.00^c^	0.00^c^	2.22^b^	< 0.01

The data presented in the table are mean ± SE. Those within a row superscripted by different letters (a, b, c) differ significantly (*p* < 0.05)

*ABWG* Average body weight gain, *ACFI* Average cumulative feed intake, *Average CFCR* Average cumulative feed conversion ratio, *D *Day

Despite the imposed experimental coccidial challenge, broilers receiving salinomycin, monensin, and thymol supplementation demonstrated body weight gains comparable to those recorded in Group A (negative control), with no statistically significant differences observed on day 42 (Table 3). These observations indicate that the inclusion of these anticoccidial agents was effective in counteracting the growth-depressing effects of coccidiosis, thereby preserving normal growth trajectories and sustaining productive performance during the post-challenge phase.

With respect to feed consumption, significant differences (*P* < 0.05) among the treatment groups became evident from day 22 onward. Broilers in Group B (positive control) exhibited significantly higher feed intake compared to Group A (negative control) and all anticoccidial-supplemented groups at day 42 (Table 3). The increased feed consumption observed in the positive control group may be indicative of compensatory feeding behavior arising from reduced feed efficiency and compromised nutrient utilization due to intestinal damage caused by coccidial infection.

Among the experimentally infected groups, broilers fed salinomycin (Group C) recorded comparatively lower feed intake along with a better feed conversion ratio (FCR), followed by those fed monensin (Group D) and thymol (Group E), when compared with the positive control group (Group B), as depicted in Table 3. This improvement in feed efficiency highlights the relative efficacy of these anticoccidial agents in maintaining intestinal integrity and optimizing nutrient utilization under coccidial challenge conditions.

### Effect on mortality

With respect to mortality, no deaths were recorded in any of the experimental groups prior to day 21. However, during the post-challenge phase, a markedly higher mortality rate (15.56%) was observed in the positive control group, whereas no mortalities occurred in the negative control or in the anticoccidial-treated groups, except for a single mortality (2.22%) recorded in the thymol-supplemented group, indicating partial protection against *Eimeria*-associated mortality compared with the positive control group.

### Effect on OPG count

The oocysts per gram (OPG) counts, as presented in Table 4, increased significantly in all experimentally challenged groups compared with the negative control group. At 7 days post-infection (dpi) (*P* < 0.05), thereby confirming the successful establishment of coccidial infection. Significant differences (*P* < 0.05) were observed in OPG values of the anticoccidial-treated groups when compared with the positive control group. In all treatment groups, OPG counts showed a marked increase at 7 dpi (*P* < 0.05); however, a progressive and pronounced decline in oocyst shedding was observed thereafter. By 14 and 21 dpi, oocyst counts were reduced to zero in broilers receiving ionophore anticoccidials (*P* < 0.001), indicating effective suppression of *Eimeria* replication and subsequent oocyst excretion. A value of zero OPG indicates that, no *Eimeria* oocysts were detected within the detection limit of the McMaster chamber during repeated microscopic examinations of the respective samples. Thymol supplementation resulted in markedly reduced oocyst shedding by 21 dpi (50 OPG), although complete suppression of oocyst excretion was not achieved, unlike the salinomycin- and monensin-treated groups. Conversely, in the positive control group, OPG counts continued to increase progressively throughout the observation period, reflecting uncontrolled parasite multiplication in the absence of anticoccidial intervention.

**Table 4:** Result of oocyst per gram of faeces (OPG) count

Treatment	OPG 21 day	OPG 28 day (7-dpi)	OPG 35 day (14-dpi)	OPG 42 day (21-dpi)
A (NC)	0 ± 0	0^b^ ± 0	0^c^ ± 0	0^b^ ± 0
B (PC)	0 ± 0	166.67^a^ ± 33.33	5783.33^a^ ± 389.37	45833.33^a^ ± 4284.99
C (Salinomycin)	0 ± 0	170^a^ ± 30.11	0^c^ ± 0	0^b^± 0
D (Monensin)	0 ± 0	180^a^ ± 35.78	0^c^ ± 0	0^b^ ± 0
E (Thymol)	0 ± 0	116.67^a^ ± 47.73	2100.00^b^ ± 296.65	50.00^b^ ± 22.36
*P* value	NS	*P* < 0.05	*P* < 0.001	*P* < 0.001

The data presented in the table are mean ± SE. Those within a column superscripted by different letters are significantly different (*p* < 0.05)

*NS* non-significant, *OPG* oocyst per gram of faeces, *NC* Negative control, *PC* Positive control

### Intestinal morphology

**Fig. 1:**
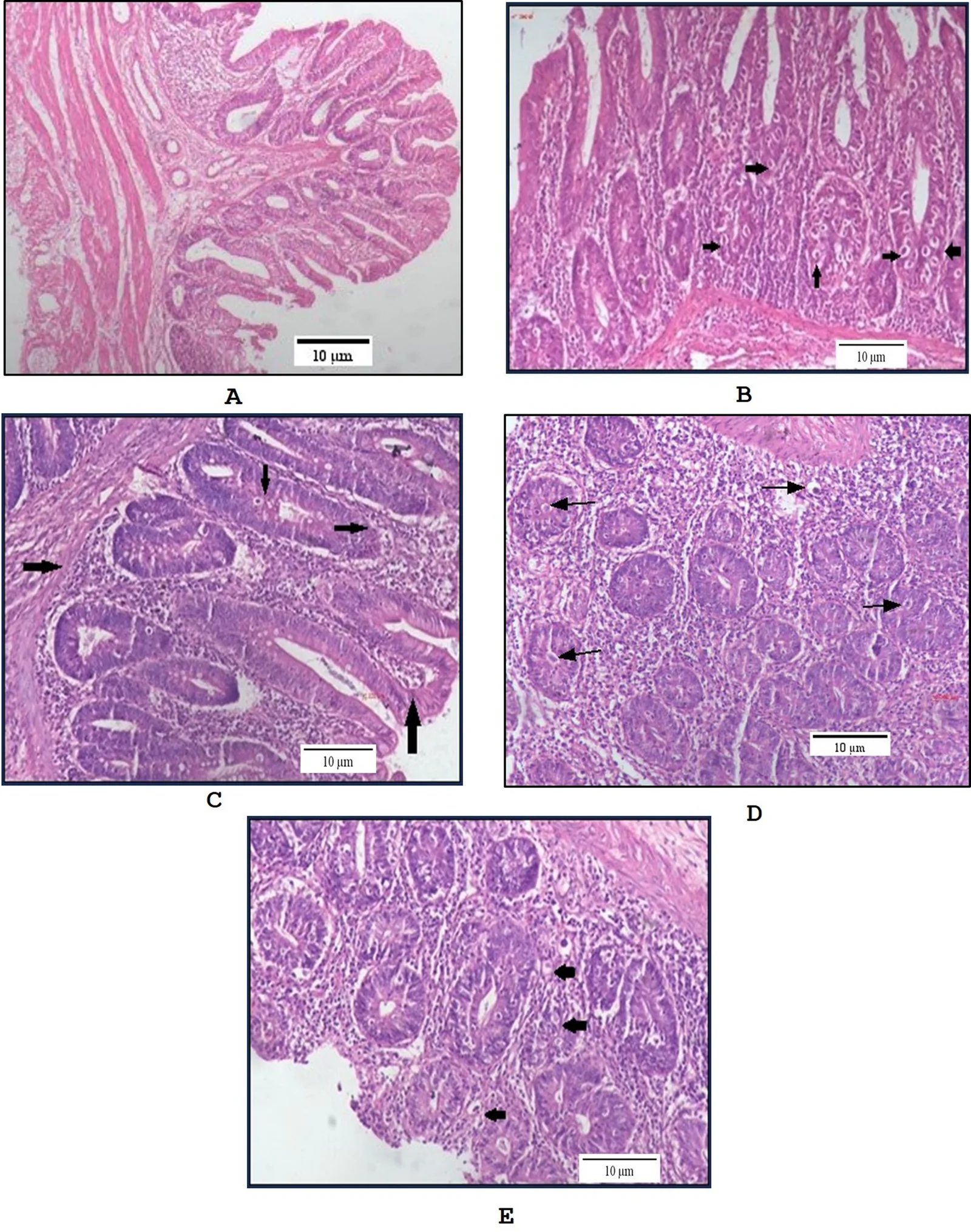
Histopathological section of cecum stained with H&E stain showing developmental stages (oocysts, trophozoites) of *Eimeria* species in the cytoplasm of enterocytes on 28th day (total magnification 200X) Except Negative control(group A). **A**: Negative control (group A) showing normal cecal histoarchitecture with intact mucosal epithelium and absence of *Eimeria* developmental stages.; **B**: Positive control (group B) showing numerous oocysts and few trophozoites; **C**: Salinomycin group (Group C) showing fewer developmental stages (oocysts); **D**: Monensin group (Group D) showing fewer developmental stages (oocysts); **E**: Thymol (group E) showing few developmental stages, including numerous trophozoites with characteristic peripheral granules and few oocysts of *Eimeria* species in the cytoplasm of enterocytes in the cecum. Scale bar = 10 μm

Histopathological examination was done at 7-day post infection, where the caecum was highly affected. Among the intestinal tissues, pathognomic changes were negligible in the duodenum, jejunum and ileum. Although *E. acervulina* and *E. mitis* were identified in the mixed inoculum, pathognomonic lesions in the duodenum, jejunum, and ileum were minimal. Consequently, the severe caecal pathology induced by *E. tenella* predominated, whereas lesions in other intestinal segments remained limited or subclinical at the time of examination. Further examination revealed significant lesions in the caecum of positive control group birds, showing extensive parasitic development, characterized by numerous oocysts and trophozoites, reflecting uncontrolled replication of *Eimeria* spp. and causing disruption of villous epithelium, necrosis of crypts, inflammatory cells in the submucosa and various developmental stages of *Eimeria* parasite (Fig. 1B). In contrast, the groups fed with ionophores and thymol showed similar but less severe histological lesions (Fig. 1C-E). Among all the infected groups, salinomycin and monensin fed group has effectively inhibited the developmental stages of *Eimeria* parasite and reduced the intestinal lesions (Fig. 1C, D). However, it has been observed that, thymol administration has not only inhibited the developmental stages of *Eimeria* but also improved intestinal histomorphology and reduced the severity of lesions when compared to the positive control group at 7-dpi (Fig. 1B and E). These observations clearly demonstrate that, while all three treatments alleviated *Eimeria*-induced intestinal damage, thymol was effective in reducing both parasite burden and host inflammatory response; however, its protective effect was comparatively lower as compared to salinomycin and monensin.

**Fig. 2:**
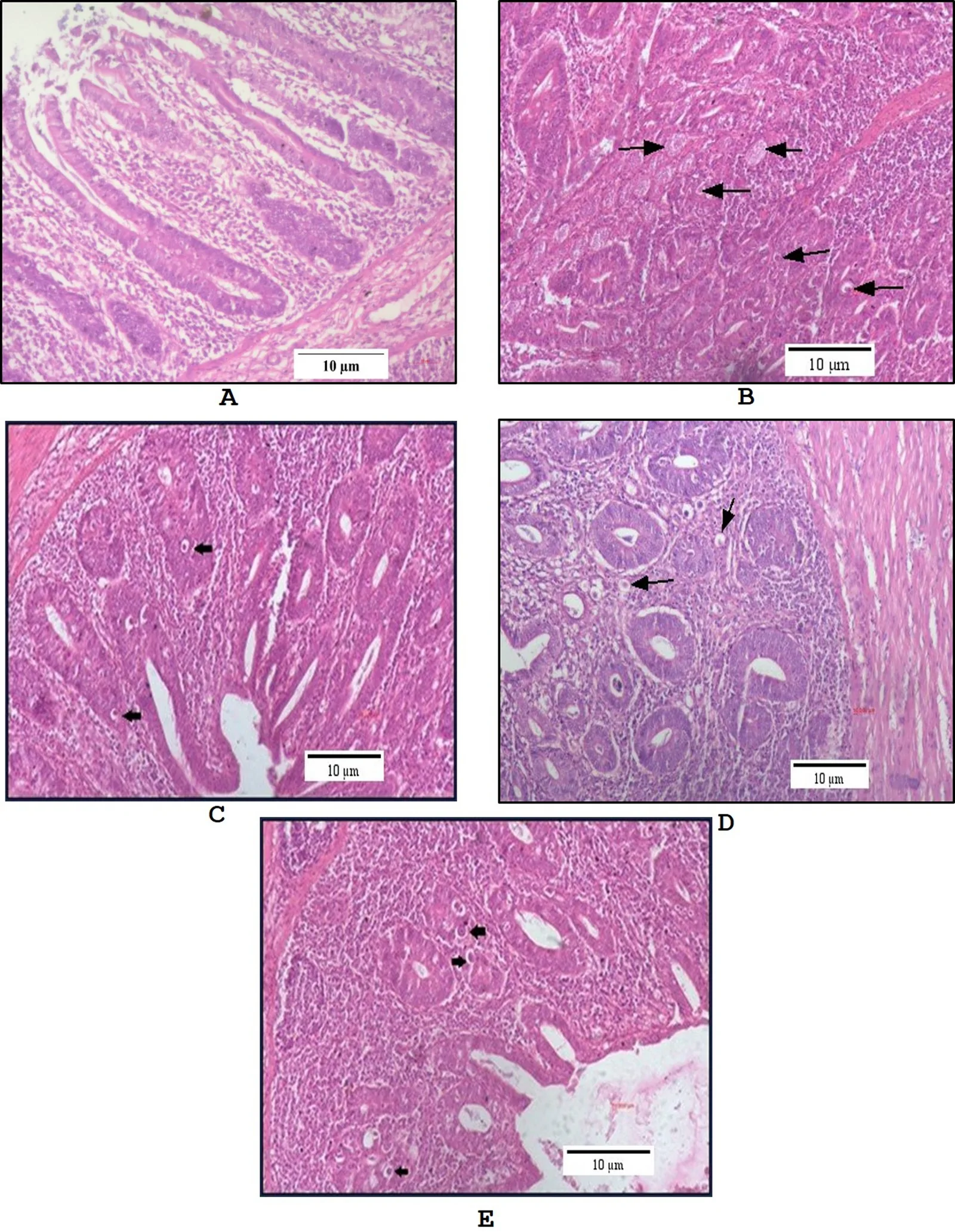
Histopathological section of cecum stained with H&E stain showing developmental stages (oocysts, trophozoites) of *E. tenella* in the cytoplasm of enterocytes on the 42^nd^ day (total magnification 200X). Except Negative control (group A); **A**: Negative control (group A) showing normal cecal histoarchitecture and absence of *Eimeria* developmental stages. Mild physiological lymphoid cells are present within the lamina propria; **B**: Positive control (Group B) showing developmental stages (numerous oocysts and few trophozoites); **C**: Salinomycin group (Group C) showing very few developmental stages (oocysts) of *E. tenella*; **D**: Monensin group (Group D) showing fewer developmental stages (oocysts) of *E. tenella*; **E**: Thymol (group E) showing minimal infiltration of inflammatory cells with very few developmental stages. Scale bar = 10 μm

At 21-dpi, the positive control group showed severe coccidial infection with numerous developmental stages of *Eimeria* spp. including abundant oocysts and some trophozoites within enterocytes, accompanied by pronounced disruption of the mucosal architecture, indicating active and advanced parasitic multiplication (Fig. 2A). In contrast, the salinomycin-treated group exhibited a substantial reduction in parasitic load, with only a few developmental stages and relatively preserved the tissue integrity (Fig. 2C). The thymol-treated group demonstrated the most pronounced protective effect, with very few parasitic stages, minimal inflammatory cell infiltration, and largely intact mucosal lining (Fig. 2E). Overall, while salinomycin effectively suppressed intracellular development of *E. tenella* compared to the positive control, thymol showed superior efficacy by markedly limiting parasite development and minimizing tissue damage and inflammation.

### Effect on relative cytokine expression

**Fig. 3:**
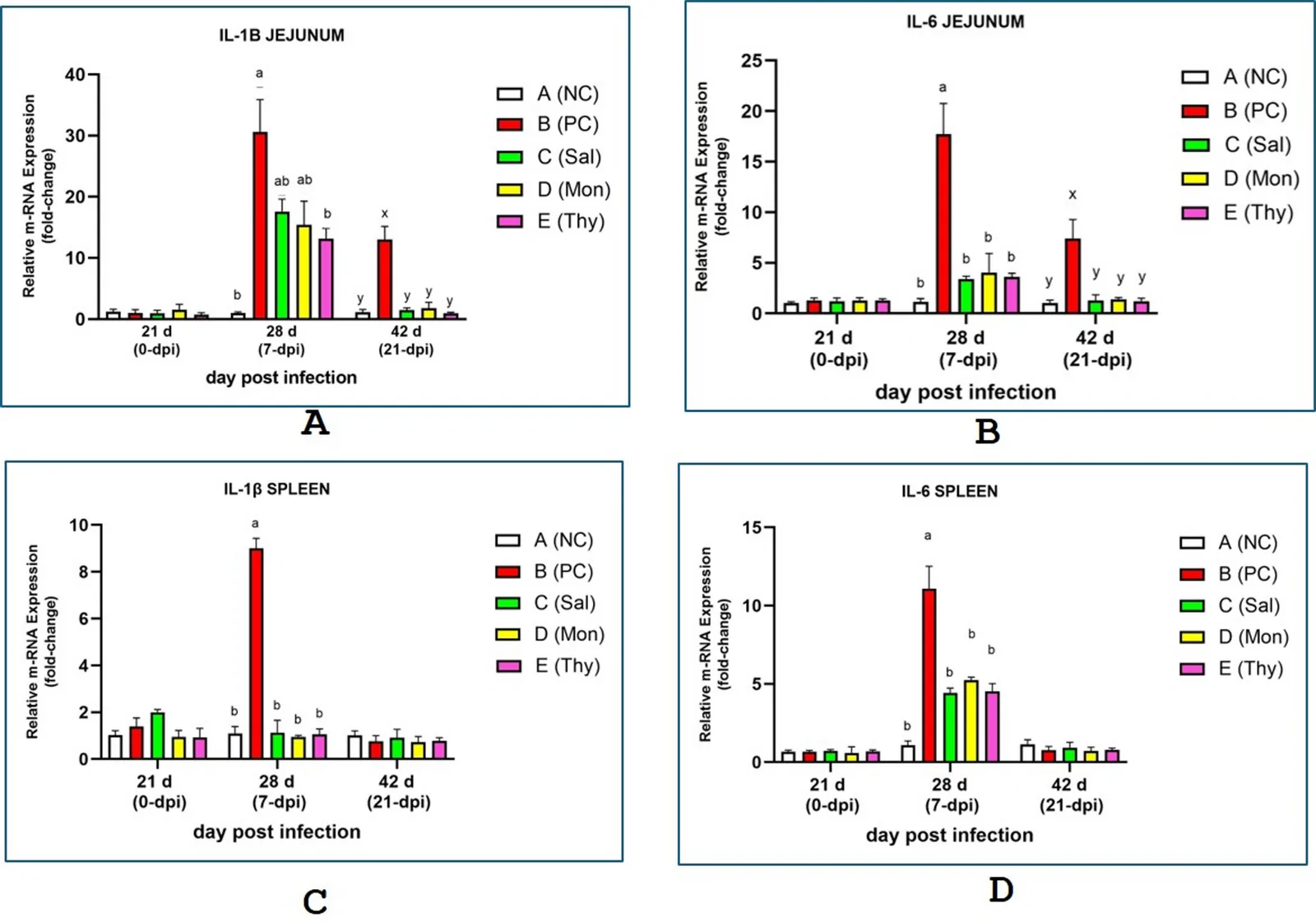
Relative mRNA expression of IL-1β and IL-6 genes in jejunal and splenic tissues of broiler chickens following challenge with mixed *Eimeria* species. **A** IL-1β expression in the jejunum; **B** IL-6 expression in the jejunum; **C** IL-1β expression in the spleen; and **D** IL-6 expression in the spleen. Expression levels were normalized against β-actin and calculated using the 2−ΔΔCt method, with the negative control group (Group A) used as the calibrator. **A** (NC): Negative control; **B** (PC): Positive control; **C** (Sal): Salinomycin-treated; **D** (Mon): Monensin-treated; **E** (Thy): Thymol-treated; d- day; dpi-day-post infection. Data are presented as Mean ± SE. Bars bearing different superscript letters differ significantly (*P* < 0.05)

Both jejunal and splenic tissues exhibited significant differences in the relative expression levels of IL-1β and IL-6 among treatment groups compared with the positive control group, as shown in (Fig. 3A–D). On day 7 post-infection, IL-1β expression was significantly higher in the positive control group than in all supplemented groups (*P* < 0.05). Among the supplemented groups, IL-1β expression was highest in the salinomycin-treated group, followed by the monensin-treated and thymol-treated groups, with thymol exhibiting the lowest expression level (Fig. 3A, C).

## Discussion

The present study aimed to compare thymol’s efficacy with commonly used ionophore antibiotics and for inter-comparison of the salinomycin and monensin. Data generated showed that broilers supplemented with salinomycin developed comparatively low inflammation-mediated pathogenesis and faster oocyst control in comparison to monensin and thymol-fed chickens. The birds of the positive control group showed increasing OPG counts from 7-dpi, with peak oocyst shedding at 21-dpi, whereas groups treated with salinomycin and monensin had a slight increase at 7-dpi followed by zero OPG counts at 14- and 21-dpi. Treatment with both ionophores demonstrated a significant decrease in OPG count equivalent to zero during the fifth and sixth weeks compared to the positive control group (Table 4). Similar findings were reported by indicating lower oocyst shedding in ionophore antibiotic-treated groups [3, 34, 35]. The OPG count in the thymol group exhibited lower oocyst shedding than positive control at 7 days post-infection, followed by a marked increase at 14-dpi and declined significantly in the last week (21-dpi) indicating appreciable anticoccidial activity. However, residual oocyst excretion was still detected, suggesting that thymol was less effective than the ionophore anticoccidials in achieving complete suppression of parasite replication under the conditions of the present study. The various factors, such as thymol dosage, infection day and route, environmental conditions, and management practices, may contribute to outcome variations as observed in other related studies [36, 37].

Although *E. acervulina* and *E. mitis* were identified in the mixed inoculum based on morphological characteristics, their relative abundance was not quantified. Therefore, these species may have been present at lower proportions than *E. tenella*, which is considerably more pathogenic and predominantly affects the caeca. Furthermore, *E. mitis* is generally regarded as a mildly pathogenic species that often produces limited or subclinical intestinal lesions. The timing of tissue collection (7 dpi) may also have influenced lesion detection, as intestinal damage caused by the less pathogenic species may have been minimal or partially resolved at the time of histopathological examination. Consequently, severe caecal pathology induced by *E. tenella* predominated, whereas lesions in the duodenum, jejunum, and ileum remained limited or subclinical.

In drug-treated groups, no significant differences were observed in body weights, feed consumption, and FCR till 21st day. However, on the 28th, 35^th,^ and 42nd day, significant differences were observed in average feed consumption and FCR of salinomycin, monensin, and thymol-treated groups, respectively. Similar findings were reported by several other research groups who observed significant weight gains on supplementation with salinomycin [30, 37–39]. During the mid-phase of the trial, feed intake decreased in all challenged groups, which was most likely due to the production of pro-inflammatory cytokines. However, in the last phase, the downregulation of cytokines might have contributed to increased appetite, resulting in similar body weights among all groups except the positive control. Regarding FCR, significant differences were observed in the later phase, with the positive control group having the poorest FCR compared to the negative control and other treatment groups (Table 3).

Ionophores (salinomycin, monensin) and thymol-fed birds developed low inflammatory response against *Eimeria* infection as reflected by reduction in the intestinal invasion by different *Eimeria* spp, downregulation in IL-1β, IL-6 production, a marked decrease in the intestinal leucocyte infiltration and reduced parasite developmental stages in comparison with the positive control group. These findings coincide with the reports of Johnson [40] and McDougald [41]. In the histopathological examination of caecal tissues of infected birds, different developmental stages of *Eimeria* spp. were observed at 7-dpi and 21-dpi. Despite supplementation with anticoccidial agents, fewer developmental stages of *Eimeria* were observed in birds treated with monensin and thymol at 21-dpi. However, very few developmental stages were demonstrated in the salinomycin-treated group in comparison to the positive control group. These findings were consistent with the results of McDougald [41], where salinomycin significantly reduced intestinal lesions caused by *Eimeria* spp. when compared to other ionophoric anticoccidial drugs.

Our study concludes that, the inclusion of thymol in feed resulted in improvement in both growth performance and intestinal health in comparison with the infected control birds. These findings coincide earlier observations [42], where it alleviates oxidative stress at the cellular level, and contributes in improvement of intestinal health. Due to its anti-inflammatory properties and effect on *Eimeria* developmental stages, thymol can provide a protective effect on intestinal mucosa during poultry coccidiosis as supported by previous findings [15, 43, 44]. However, the thymol-treated group showed higher OPG counts as compared to other ionophore antibiotic-treated groups, indicating comparatively low potency to existing anti-coccidial drugs. Therefore, it is more sensible to use thymol as an alternative approach to reduce the incidence of anticoccidial resistance.

## Conclusion

Among the ionophores evaluated, salinomycin and monensin showed comparable anti-coccidial efficacy by completely suppressing oocyst shadding at 14 and 21 dpi, improving intestinal histopathology, and attenuating inflammatory cytokine expression whereas Supplementation of thymol exhibited beneficial immunomodulatory and histopathological effects but comparatively lower anti-coccidial and anti-inflammatory properties, suggesting an effective alternative to the chemical anticoccidial drugs. However, the comparatively delayed reduction in oocyst shedding limits its utility under acute infections. Lack of molecular confirmation of the *Eimeria* species and use of mixed species infection model preclude species-specific elucidation of thymol response. Further studies involving molecular characterization and experimentally controlled single-species infection are required to determine the precise anti-coccidial efficacy of thymol.

## Supplementary Information

Supplementary Material 1.

Supplementary Material 2.

## Data Availability

All data generated or analyzed during this study are available from the corresponding author on reasonable request. Supporting data are presented in the Supplementary Materials.

## Abbreviations

ABWG: Average Body Weight Gain
ACFC: Average Cumulative Feed Consumption
BIS: Bureau of Indian Standards
cDNA: Complementary Deoxyribonucleic Acid
dpi: Days Post Infection
FCR: Feed Conversion Ratio
H&E: Hematoxylin and Eosin
IL-1β: Interleukin-1 beta
IL-6: Interleukin-6
K₂Cr₂O₇: Potassium Dichromate
mRNA: Messenger Ribonucleic Acid
NCBI: National Center for Biotechnology Information
OD260/OD280: Optical Density at 260/280 nm
OPG: Oocysts Per Gram (of feces)
PCR: Polymerase Chain Reaction
ppm: Parts Per Million
qPCR: Quantitative Polymerase Chain Reaction
qRT-PCR: Quantitative Real-Time Polymerase Chain Reaction
RNA: Ribonucleic Acid
rRNA: Ribosomal Ribonucleic Acid
RT: Reverse Transcription
