# Supplementary material for: The comparative effect of ionophore and thymol on growth performance, intestinal histopatholgy and cytokine expression in broilers challenged with mixed Eimeria species
Source: BMC Vet Res. 2026 Aug 6;22:663. doi: 10.1186/s12917-026-05794-0 (PMC13733107; doi:10.1186/s12917-026-05794-0)
Supplement: Supplementary file 1 [file 12917_2026_5794_MOESM1_ESM.docx]

**Supplementary figures**

**Fig. S1**

**A**

**Fig. S1:** Result of interview schedule designed for poultry farmers regarding use of anti-coccidial drugs at their poultry farm. **A:** Summarized responses collected from poultry farmers regarding the use of anticoccidial drugs across different poultry production systems; **B:** Distribution of poultry farms based on bird type; **C:** Commonly used anticoccidial drugs in poultry farms; **D:** Most frequently used anticoccidial drug; **E:** Mode of administration of anticoccidial drugs; **F:** Drug rotation patterns followed in poultry farms; **G:** Farmer perception of most effective anticoccidial drug; **H:** Occurrence of coccidiosis outbreaks despite drug usage; **I:** Source of recommendation for anticoccidial drug use; **J:** Reason for using particular anticoccidial agent.
