# Supplementary material for: The comparative effect of ionophore and thymol on growth performance, intestinal histopatholgy and cytokine expression in broilers challenged with mixed Eimeria species
Source: BMC Vet Res. 2026 Aug 6;22:663. doi: 10.1186/s12917-026-05794-0 (PMC13733107; doi:10.1186/s12917-026-05794-0)
Supplement: Supplementary file 2 [file 12917_2026_5794_MOESM2_ESM.docx]

**Fig. S2**

**Fig. S2:** Morphological identification of different *Eimeria* species used in the study done by using calibrated binocular research microscope by taking the mean values of measurements and shape index (length/width ratio) was calculated. The average shape index (*E. mitis*- 1.04), (*E. tenella*-1.15), and (*E. acervulina*-1.22). **A**: *Eimeria mitis* Sporulated oocysts (Shape index: 1.07); **B**: *Eimeria mitis* Sporulated oocysts (Shape index: 1.05); **C**: *Eimeria tenella* Sporulated oocysts (Shape index: 1.15); **D**: *Eimeria tenella* Sporulated oocysts (Shape index: 1.16); **E**: *Eimeria acervulina* Sporulated oocysts (Shape index: 1.23); **F**: *Eimeria acervulina* Sporulated oocysts (Shape index: 1.20)
